# Balancing the Risk: Heritability of Leaf Phenology and Frost Resistance in the Dominant Temperate Forest Tree European Beech

**DOI:** 10.1002/ece3.74080

**Published:** 2026-07-25

**Authors:** Jonas Schmeddes, Niels Preuk, Ilka Beil, Eike Marie Dittmar, Andrey Malyshev, Annè Lemke, Charlotte Loft, Dennis Quadt, Maximilian Richardt, Tom Woweries, Juergen Kreyling

**Affiliations:** ^1^ Institute of Botany and Landscape Ecology, Experimental Plant Ecology University of Greifswald Greifswald Mecklenburg‐Vorpommern Germany

## Abstract

Ongoing climate change is increasingly affecting forest ecosystems. European beech, the naturally dominant tree species of Central Europe, has suffered in recent dry years. Due to its frost sensitivity and conservative spring leaf out behavior, it is also less able to profit from generally prolonged growing seasons due to climate warming, compared to other species. Its potential to maintain its competitiveness depends on its capacity to adapt to the changing environmental conditions; otherwise, the species might lose its dominance and face shifts in its distribution range. Generally, the capacity to adapt by selection depends on phenotypic variation of traits and their heritability in a population or species. Here, we aimed to quantify heritability of a set of relevant phenological and physiological traits. Based on 961 half‐sib seedlings, originating from mother trees with early, intermediate, and late leaf out from a natural forest stand in north‐east Germany, we measured spring (leaf out date) and autumn (leaf senescence) phenology as well as the frost tolerance of buds in late winter and of leaves in spring. Narrow‐sense heritabilities were calculated by animal models and half‐sib analysis. The European beech seedlings revealed a medium heritability in spring phenology (*h* ^2^: 0.48 and 0.57 for two consecutive years) and autumn leaf senescence (*h* ^2^: 0.35). The physiological traits leaf and bud frost tolerance had a low heritability (*h* ^2^: 0.13; *h* ^2^: 0.12, respectively). This demonstrates a considerable capacity to adapt spring and autumn phenology if the selective pressure increases. The possibility of a paralleled adaptive evolution of frost tolerance, however, remains questionable.

## Graphical abstract

We quantified the heritability of spring and autumn phenology and frost tolerance in European beech using a common‐garden experiment with 961 seedlings from half‐sib families. Spring leaf out and autumn senescence showed moderate heritability, indicating substantial adaptive potential, whereas bud and leaf frost tolerance exhibited low heritability. These results suggest that while phenological timing may evolve under climate change, adaptive responses in frost tolerance may be constrained.

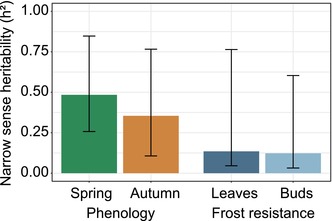

## 1 Introduction

Long‐lived, sessile organisms such as trees require effective mechanisms to cope with changing environmental conditions. Populations can respond to environmental change through phenotypic plasticity, range shifts, or genetic adaptation—or combinations thereof (Aitken et al. 2008; Hoffmann and Sgrò 2011). While range shifts and plasticity have received considerable attention, the potential for in situ genetic adaptation is often underappreciated in trees (Alberto et al. 2013). Genetic adaptation through natural selection requires heritable trait variation: narrow‐sense heritability (*h* ^2^), defined as the proportion of phenotypic variance attributable to additive genetic effects (Falconer and Mackay 1996), determines the rate at which a population can respond to directional selection via the breeder's equation (Alberto et al. 2013). High heritability thus facilitates rapid adaptive responses, whereas low heritability implies that even strong selective pressures result in only slow or limited evolutionary change. A further prerequisite for adaptation is sufficient phenotypic variation among individuals—a “selective window” in which fitness differences between individuals arise and selection can act. Importantly, the pace of adaptive evolution also depends on the strength and consistency of selection, which differs among traits (Kingsolver et al. 2001). Ongoing climate change exerts novel and potentially strong selective pressures on tree populations (Hoffmann and Sgrò 2011; Alberto et al. 2013), making the assessment of heritability of climate‐relevant traits a key step towards understanding whether and how fast species can adapt (Aitken et al. 2008). Here, we therefore quantify the heritability of phenological and physiological traits in a central European forest tree species exposed to changing selection regimes.

The timing of phenological events, especially leaf out and leaf senescence, is highly important for deciduous trees. They determine the duration of the trees' photosynthetically active period and are key traits for the plant's fitness (Chuine 2010). Both phenological traits, leaf out and leaf senescence, are induced by environmental cues and therefore vary in their timing. Photoperiod, temperature, and water availability are the main cues for leaf out and leaf senescence (Vitasse et al. 2009; Fu et al. 2015). Further, both leaf out and leaf senescence are also known to influence each other's timing (Beil et al. 2021; Fu et al. 2014).

Plant growth is often temporally restricted by cold temperatures or droughts. Plants in seasonal climates maximize their growing season by leafing out as early as possible and shedding as late as possible. However, during leaf unfolding and cessation, frost represents a major risk for the plant. During leaf unfolding, late spring frost events can severely damage leaves and reduce the tree's fitness (Muffler et al. 2024; Baumgarten et al. 2023). Affected trees need time and resources to recover, impairing their growth (Príncipe et al. 2017). Similarly, early autumn frost events that occur during leaf senescence can affect a plant's fitness by hampering the retraction of nutrients from the leaf, potentially leading to an early leaf cessation (Norby et al. 2003; Fracheboud et al. 2009; Hänninen et al. 1990; Estiarte and Peñuelas 2015). Trees facing competition for light or environments with summer droughts benefit from an early start of growth, as spring and early summer often provide warmer, more favorable conditions with climate change. (Kramer et al. 2000; Robson et al. 2013; Giagli et al. 2016).

The leaf out date is known to be a heritable trait, demonstrated for many deciduous trees and also for the target species of this study, *Fagus sylvatica* , European beech (Gauzere et al. 2016; Westergren et al. 2023; Kramer et al. 2008; Frank et al. 2017; Bontemps et al. 2016). However, the occurrence of late spring and early autumn frost events is hardly predictable (Zohner et al. 2020; Lamichhane 2021). Under such unpredictable conditions, there are different phenological strategies with different risk–benefit trade‐offs (Xin 2016). Depending on the timing and severity of frost events, it can be advantageous to leaf out or shed leaves earlier or later (Xin 2016; Baumgarten et al. 2023). Trees with an early leaf out strategy could achieve a higher carbon gain under climate warming conditions but are more likely to be damaged by late frost events than late flushing trees. However, if frost damage occurs, early flushing trees have more time for recovery as they started early into the growing season (Menzel et al. 2015; Baumgarten et al. 2023).

Besides frost avoidance, frost tolerance is another strategy to cope with low temperatures. Some experiments have demonstrated significant phenotypic variation in frost tolerance among European beech provenances (Kreyling et al. 2014; Hofmann et al. 2015), whereas Muffler et al. (2024) found no significant differences in frost tolerance among provenances. Whether frost tolerance of trees is heritable and, consequently, adaptable, is unclear. If true, also an adaptation towards earlier leaf out or later leaf senescence beyond the current physiological limits would be eased, as the phenology could evolve alongside increasing frost tolerance.

**FIGURE 1:**
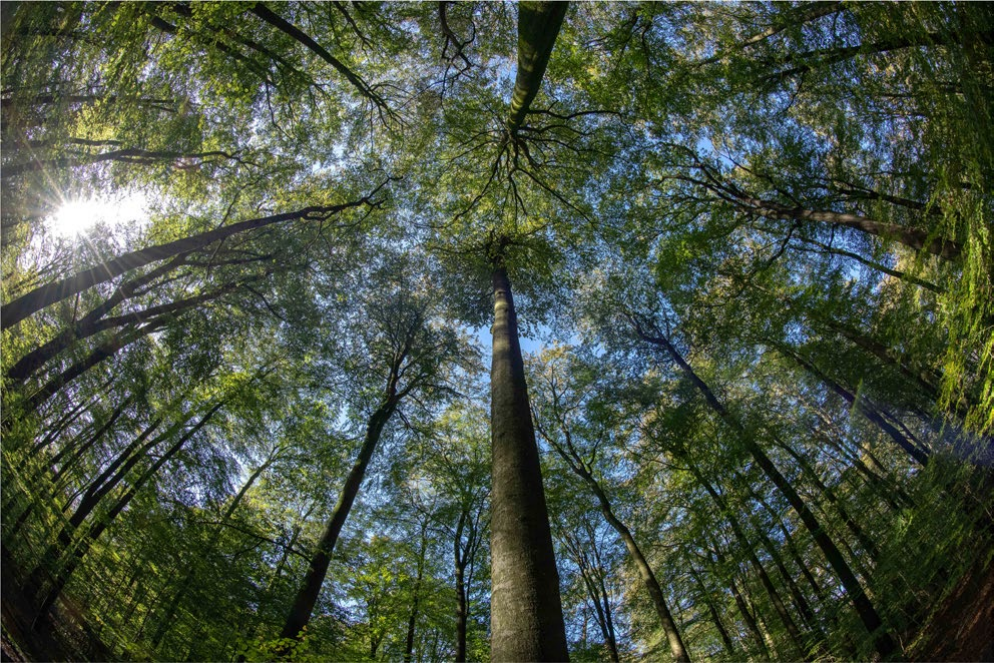
European beech ( *Fagus sylvatica* L.) is the dominant natural forest tree species of Central Europe, which forms monodominant stands such as the site containing the mother trees of this study in the National Park Müritz (forest district Serrahn, Unit 5409, 53.34° N, 13.21° E). Picture: Juergen Kreyling, 02/10/2019.

European beech is the dominant deciduous forest tree of Western and Central Europe (Figure 1; Leuschner and Ellenberg 2017) and is increasingly challenged by the consequences of climate change. European beech is considered particularly frost‐sensitive in spring (Vitasse et al. 2014). Whether European beech populations possess the adaptive potential to respond genetically to shifting frost regimes depends on two complementary strategies: frost avoidance through phenological adjustment (earlier or later leaf out and leaf senescence) and frost tolerance through physiological resistance of buds and leaves (Lenz et al. 2016). Both strategies may be subject to natural selection under increasing frost risk, but only heritable trait variation can serve as the substrate for evolutionary adaptation (Falconer and Mackay 1996; Alberto et al. 2013). We therefore hypothesized that (1) spring and autumn phenology, as traits with known population‐level differentiation and strong environmental cues, exhibit higher narrow‐sense heritability, whereas (2) frost tolerance of buds and leaves, as more constitutive physiological traits, exhibit lower heritability. To test these hypotheses, we conducted a common garden experiment with seedlings from 11 mother trees of a single population from north‐eastern Germany, selected to represent contrasting leaf‐flushing phenotypes based on 12 years of field observations (Malyshev et al. 2022). This half‐sib family design allowed us to partition phenotypic variance into additive genetic and environmental components, and thus to estimate the narrow‐sense heritability of frost avoidance and frost tolerance traits, and to assess the adaptive potential of European beech in the face of changing frost regimes.

## 2 Methods

### 2.1 Plant Material and Experimental Design

#### 2.1.1 Mother Trees

The mother trees are located in the Müritz National Park, northeast Germany (forest district Serrahn, Unit 5409, 53.34° N, 13.21° E; Figure 1). The climate is temperate with maritime influence. The European beech forest of Serrahn is characterized by a long period with comparatively little human intervention (Malyshev et al. 2022). A continuous forest cover was present since the 16th century. Since 1845, the forest has been under various types of protection and is now designated as a total reserve in the Müritz National Park (Malyshev et al. 2022). In an area of 1 hectare, 146 canopy trees of *Fagus sylvatica* have been under observation of spring phenology since 2008 (see Malyshev et al. 2022 for details) by the National Park Service. The trees were categorized by their mean leaf out dates as early, intermediate, or late‐flushing. Out of these groups, 11 trees (four early, two intermediate, five late) were randomly selected as mother trees for beechnuts collection. Over the 12‐year period, the early flushing trees leafed out on average 4–5 days earlier than the median tree, while late flushing trees leafed out 4–10 days later.

#### 2.1.2 Collection and Cultivation of the Offspring

In September 2019, beechnuts were harvested directly from each tree with a slingshot (Bigshot, Sherrill Inc). In October, they were planted individually in pots of 500 mL volume in a substrate consisting of equal parts of sandy loamy topsoil, compost, and sand. The seedlings were grown under outdoor conditions at the university campus in Greifswald (54.0919° N, 13.3647° E, approx. 100 km north‐east from the mother trees site). The seedlings were irrigated as needed and shaded from May to October using a 65% shade net. To ensure comparable microclimatic conditions, their positions were rotated weekly. Over winter, the pots were buried to their brim in sand to protect the roots from lateral frost. In March 2021, the seedlings were fertilized using an organic fertilizer (1.5 g Oscorna Animalin).

### 2.2 Measurement of Quantitative Traits

#### 2.2.1 Spring and Autumn Phenology

In the spring of 2021, we monitored spring phenology three times a week at the common garden in Greifswald. The leaf out date was defined as the day on which the first leaf had fully unfolded and the petiole was visible. Spring phenology was observed in 2021 on 922 seedlings of the 11 mother trees.

In autumn 2021, we tracked leaf senescence on 403 seedlings of 11 mother trees by weekly greenness measurements using a SPAD 502 Plus Chlorophyll Meter. We measured SPAD values on five leaves per tree that were representative of the overall senescence level of the individual tree at that date. In order to track the last phase of senescence, leaves that had completely turned brown or abscised were set to 0. We then calculated the day of 10%, 50%, and 90% senescence in relation to the mean SPAD values during September by linear interpolation between the measurement dates. However, only 50% senescence was used for further analysis, as the 10% and 90% senescence values are more likely influenced by noise in the measurements and are presumably less representative.

During the winter 2021/2022, 592 seedlings from all mother trees were planted in randomly mixed groups of 37 seedlings into 16 containers for further long‐term observation, with half of the seedlings per container stemming from the early 50% of all seedlings and half from the late 50% of the seedlings, based on their phenology in spring 2021. The containers had a volume of 65 L, were filled with the same substrate mixture as the pots before (see above), and had drainage holes in the bottom. Spring phenology was observed in 2022 with identical methodology as described above for 2021 on the surviving 562 seedlings of the 11 mother trees.

#### 2.2.2 Bud and Leaf Frost Tolerance

We returned a subset of seedlings not used for the phenological observations described above to the site of the mother trees in the Müritz National Park during winter, in order to expose the offspring to the same natural conditions as the mother trees. This design was intended to allow for a calculation of heritability by parent‐offspring regression, a method for which parents and offspring need to experience the exact same environment. However, alternative methods (Animal Model, half‐sib analysis, see below for detail) were ultimately used for analysis. We randomly chose 11 seedlings from each mother tree, resulting in a total of 121 seedlings. We randomly placed one offspring of each mother tree in a box of 3 × 4 pots and repeated this for 11 boxes, with the twelfth pot containing no plant but a temperature logger (“TMS‐4” by TOMST using their software “lolly 1.32”), which was used to log air temperature for each box. The soil surface of the pots was covered with dead leaves for mimicking natural insulation, while the room between the pots and 5 cm around the box edge (bottom and sides) was filled with insulation foam to protect the soil in the pots from lateral frost.

After leaf senescence was completed (09/11/2021), one box containing one offspring of each mother tree was fastened in the canopy of each mother tree, respectively. This was accomplished by using a rope, thrown over a branch, to pull up the respective box attached on the other end of the rope. In addition, there was a rope around the box which was attached to the trunk of the mother tree to keep the box as steady as possible. The boxes were placed at the same height as the buds of the adult trees (20–30 m).

Towards the end of winter (24/02/2022), the boxes with the offspring were collected again, together with freshly cut twigs of the individual mother trees, for bud sampling. The offspring and the twig samples were brought directly to the lab at the University of Greifswald. Here, the twigs of the mother trees were freshly cut and wrapped in a wet cloth and a plastic bag. Together with the offspring‐boxes they were stored outside in the shade until their bud frost tolerance was measured three times per individual, with one measurement each day within 3 days, respectively. We did not observe relevant changes in frost tolerance due to the storage as temperatures during storage were similar to those previously experienced in the forest. Note, though, that frost tolerance measurements of the mother trees were only used for illustrative purposes and that the heritability calculation was exclusively based on the offspring, which all experienced identical conditions during storage. Due to storm Zeynap (19/02/2022), the box on tree B3 was hanging at a height of 5 m and the boxes on the trees A2, S2, U2, and Q41 had completely fallen down. We do not expect this event to have substantially affected the results, since it happened just 5 days before final sampling and affected all seedlings of the boxes evenly, that is, one offspring of each mother tree per box. Moreover, the thermometer in the box on tree B3 had fallen out, whereas the thermometer in the box on tree S2 went missing following the storm. However, the offspring did not show any visible damage and were therefore included in the further analysis and the measured temperatures also were comparable.

Based on the measured air temperatures, we also calculated freezing degree days and growing degree days per box (Table 1). Growing degree days are defined as the sum of days over a baseline of 5°C. Freezing degree days are defined as the sum of days below a baseline of 0°C. Data were processed using the myClim package for the R statistical environment (Man et al. 2023).

**TABLE 1:** Air temperature characteristics for the frost experiment measured in the seedlings' boxes within the mother trees' canopies from 09/11/2021 to 24/02/2022. Growing degree days are defined as the sum of days over a baseline of 5°C. Freezing degree days are defined as the sum of days below a baseline of 0°C. Tree S2 is not shown as the temperature logger was lost.

Mother tree ID	Mean (°C)	Min. (°C)	Max. (°C)	Range (°C)	Frost days	Freezing degree days	Growing degree days
A2	2.4	−4.1	10.2	4.1	51	59	53
A6	2.7	−4.5	10.6	4.0	45	55	63
B3^a^	2.4	−4.4	10.3	3.8	49	57	55
K5	2.7	−4.6	10.6	4.0	44	54	63
Q21	2.7	−4.4	10.6	4.0	45	54	63
Q41	2.8	−4.4	10.7	3.8	45	53	64
S3	2.7	−4.4	10.5	3.9	44	54	63
S5	2.7	−4.5	10.6	3.9	44	55	62
U2	2.5	−3.9	9.6	3.9	49	55	53
W1	2.9	−4.3	10.7	3.9	42	50	68

^a^ Thermometer fallen out of the seedling box, likely on 19/02/2022, affecting 5 out of 108 days of measurements.

#### 2.2.3 Bud Frost Tolerance

Frost tolerance was measured in three buds from each of 121 offspring and, for comparison, in the 11 mother trees. In three runs, buds were randomly selected, with each tree sampled once per run. Intact lateral buds of approximately the same size were used. In a very few cases where no lateral bud was available, a terminal bud was sampled. Frost tolerance was determined using the DTA method (differential thermal analysis) according to Malyshev et al. (2020). DTA was chosen for its high throughput and quantitative resolution. European beech buds produce a well‐defined low‐temperature exotherm (LTE) during controlled freezing, confirming the method's suitability for this species and tissue type (Neuner et al. 2019; Malyshev et al. 2020). Unlike electrolyte leakage, it requires no incubation, preset temperature levels, and interpolation and allows overnight processing of large sample numbers with minimal preparation. Miniature thermocouple (type T, Omega 5SRTC‐TT‐TI‐36‐1M) were inserted and fixed into the center of the buds, which were then cooled down from 0°C to −30°C in a freezing chamber (WT 100, Weiss Umwelttechnik GmbH). The freezing rate was −3°C per hour, consistent with established DTA protocols for woody plants in which this rate has been shown to yield reliable and reproducible LTE detection (Neuner et al. 2019; Malyshev et al. 2020). In combination with thermocouple data acquisition modules (Omega TC‐08) and PicoLog 6 as recording programme, the temperature inside the buds was measured. Before freezing, each bud was dipped in water to ensure an even surface wetness. If the bud's intracellular water freezes, this exotherm reaction can be seen in a brief rise in temperature measured inside the bud. This phenomenon is called LTE. Since freezing of intracellular water is lethal for tree cells, the timing of the LTE can be used as a measure of the frost tolerance of the studied tissue (Hänninen 2016). Therefore, the lowest recorded temperature rise defines the frost tolerance of the bud, determined from the mean temperature of the beginning and end of the rise. The temperature curve of each bud was compared with that of three control buds per run. The control buds were taken randomly from one individual and boiled for at least 10 min before freezing to ensure that their tissue was no longer alive and therefore would not show the LTE. They were used as a reference for the general temperature in the freezer chamber to ensure that only real increases due to LTE and not temperature fluctuations were evaluated. The final frost tolerance for an individual was calculated as the mean value across the three measurements per individual.

#### 2.2.4 Frost Tolerance of Freshly Unfolded Leaves

The seedlings which were used for the assessment of buds' frost tolerance were kept at the common garden facility described above and used again to measure frost tolerance of freshly unfolded leaves, again using the DTA Method according to Malyshev et al. (2020) with the same equipment as described for buds' frost tolerance above. Within 2–3 days after leaf out the second, third, and fourth uppermost leaves were sampled of each seedling. Leaf samples (approx. 2 cm^2^) were wrapped around the thermocouple sensor and fixed with tape. We used an aluminum block with holes to place the rolled leaf pieces with the attached sensor, closing the holes off with cotton wool to ensure stable temperatures around the samples. The chamber temperature was decreased from 10°C to −15°C at a rate of approx. −3°C per hour (max. −4°C per hour). The quantification of frost tolerance of the leaves was then done the same way as described above for the buds.

### 2.3 Data Analysis

We determined narrow‐sense heritability (*h* ^2^) for all traits by using generalized linear mixed effects models, also known as ‘animal models’. Animal models partition the phenotypic variation of a trait into different components, thereby allowing us to quantify the proportion of phenotypic variation due to additive genetic variation (Wilson et al. 2010). We fitted animal models using the MCMCglmm package for the R statistical environment (Hadfield 2010). In order to partition the total phenotypic variance, we built univariate models to estimate the additive genetic variance and residual variance of the respective traits. The underlying pedigree solely included the maternal relation between offspring, which means that offspring that share the same mother individual are defined as maternal half‐siblings.

Bayesian approaches such as MCMCglmm require the specification of suitable priors. We used two different sets of priors depending on the considered traits. For the phenological traits, spring leaf out and autumn leaf senescence, we applied an informative parameter expanded prior for the additive genetic variance and an informative inverse‐Wishart prior for the residual variance (de Villemereuil et al. 2013). The frost tolerance traits of buds and leaves were assigned inverse‐Wishart priors, generated by partitioning the phenotypic variance evenly among variance components, i.e., additive genetic variance and residual variance (Hadfield 2010; Taylor et al. 2002).

We ran the Markov Chain Monte Carlo algorithm (MCMC) to obtain posterior distributions of the model parameters. Each run comprised four parallel chains with 1,000,000 iterations each and a burn‐in of 200,000 iterations to foster stationarity and convergence. We additionally applied a thinning interval of 500 steps to reduce autocorrelation between samples and increase the effective sample size. We visually evaluated trace plots and density plots for chain convergence. Accordingly, chain convergence was assumed when trace plots showed no trends, that is, bending, and posterior distributions met the assumption of normality (Hadfield 2010).

We extracted the mean estimates and the 95% highest posterior density intervals from the posterior distributions of the variance components. Finally, narrow‐sense heritability was calculated from the posterior distribution of the variance components as the ratio of additive genetic variance (*V*_a_) to total phenotypic variance (*V*_p_ = additive genetic variance (*V*_a_) + residual variance (*V*_r_)).

A common half‐sib analysis was carried out for each trait for comparison to the animal model. A linear mixed model was fitted to obtain variance components, using the lme4 package in R. In the model, families were defined as random effects to account for unequal family sizes. As half sibs share only a quarter of their genes, the variance among half‐sib families reflects only ¼ of the additive genetic variance. Thus, narrow‐sense heritability was calculated as four times the among‐family variance (4*V*_family_), divided by the sum of variance among‐family (*V*_family_) and residual variance (*V*_r_). Finally, 95%‐confidence intervals were calculated, using 1000 bootstrapped replications, based on the boot package in R.

## 3 Results

**FIGURE 2:**
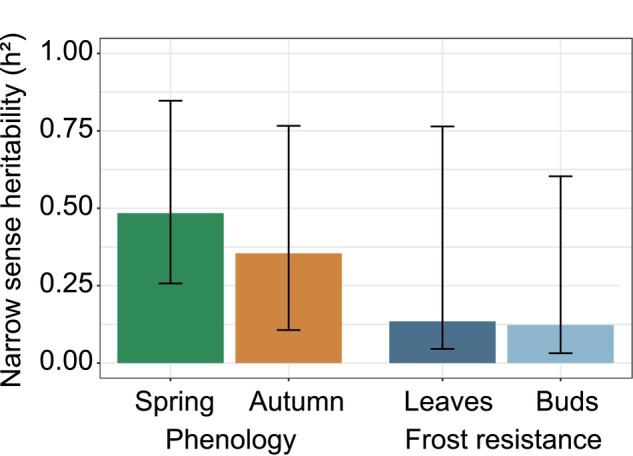
Narrow sense heritability in European beech seedlings in spring phenology (leaf out date), autumn phenology (leaf senescence), and frost tolerance of leaves and buds estimated by animal models, including 95% credible intervals.

**FIGURE 3:**
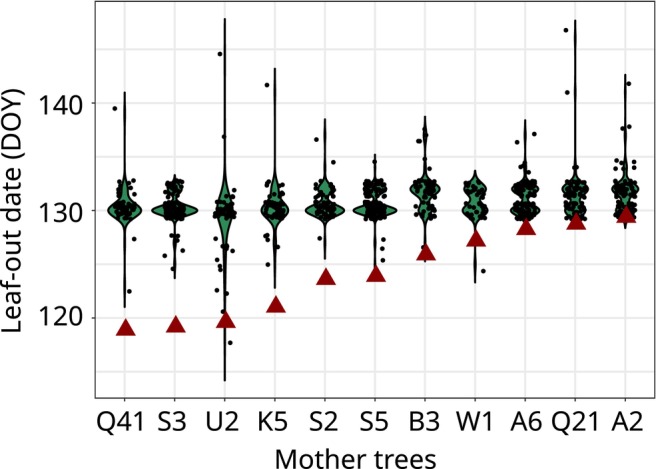
Spring phenology quantified as leaf out date of seedlings, sorted by the average leaf out dates of their mother trees (red triangles, average across 2008–2019 per mother tree in the forest stand, respectively). Dots and violin plots show the leaf out date of their offspring in the common garden in year 2021. The leaf out dates of 2022 resulted in a similar pattern. DOY, day of the year.

Spring phenology showed medium high heritability in the studied European beech seedlings (Figure 2; $h_{2021}^{2}$: 0.48; $h_{2022}^{2}$: 0.57, based on animal model). This demonstrates that a considerable proportion of trait variation can be attributed to genetic effects. This is also visible when depicting the leaf out date of the seedlings ordered by the mean leaf out date of their mother trees over 12 years (Figure 3). Differences in the spring phenology of the seedlings resemble mean spring phenology patterns of their mother trees.

Autumn phenology (leaf senescence) showed a lower but still moderate heritability (Figure 2; *h* ^2^: 0.35, based on animal model). This means that the trait variation in leaf senescence is partially explainable by genetic effects.

**FIGURE 4:**
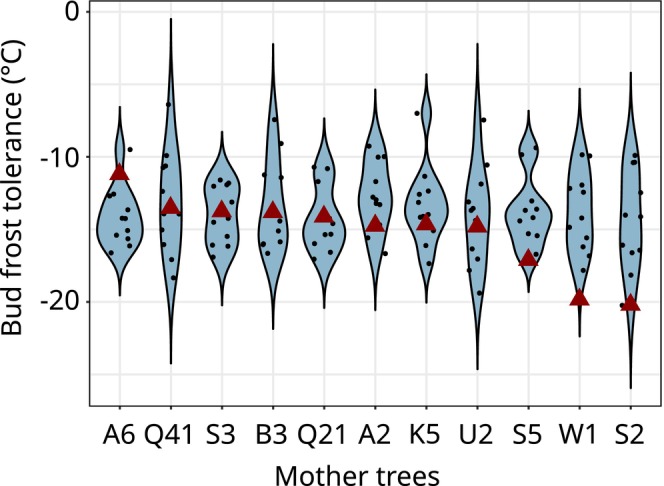
Frost tolerance of buds in late February 2022 sorted by the frost tolerance of their mother trees (red triangles). Dots and violin plots show the frost tolerance of their seedlings.

In contrast to the phenological traits, heritability of the physiological traits frost tolerance in leaves and in buds was only marginal (Figure 2; leaves: 0.13, buds: 0.12, based on animal model). This demonstrates that only a small proportion of trait variation is attributable to genetic effects (Figure 4). Also, the span in the frost tolerance in freshly unfolded leaves of the most and the least tolerant individuals was small (average: −7.76°C ± 0.72 SD).

We also calculated the heritability by the formerly common half‐sib analysis and obtained similar results as by the animal model described above. For the phenological traits, the results are in line with the results from the animal model (Table 2). For the frost tolerance traits, the maternal variance component was estimated as zero by the half‐sib analyses, indicating no detectable among‐family variation. Accordingly, narrow‐sense heritability was estimated as *h* ^2^ = 0 for both frost tolerance traits (Table 2).

**TABLE 2:** Narrow sense heritability of the studied traits comparing the results of animal models and half‐sib analyses. For the frost tolerance traits, the maternal variance component was estimated as zero by the half‐sib analyses, indicating no narrow‐sense heritability in those traits and preventing the estimation of confidence intervals.

Trait	*n*	Animal model		Half‐sib analysis	
		*h* ^2^	Credibility interval	*h* ^2^	Confidence interval
Spring leaf out 2021	922	0.48	0.26–0.85	0.52	0.30–0.74
Spring leaf out 2022	564	0.57	0.32–0.88	0.65	0.37–1.01
Autumn leaf senescence	403	0.35	0.11–0.77	0.33	0.12–0.55
Leaf frost tolerance	83	0.13	0.05–0.76	0.00	NA
Bud frost tolerance	112	0.12	0.03–0.60	0.00	NA

## 4 Discussion

We found a substantial heritability in both spring ($h_{2021}^{2}$: 0.48; $h_{2022}^{2}$: 0.57) and autumn phenology (*h* ^2^: 0.35), estimated by the animal model. The half‐sib analysis further corroborated these results, however, we decided to base the discussion primarily on the results of the animal model, as we assume it to be the more advanced and reliable approach.

Previous studies vary strongly in reported heritabilities in spring phenology of European beech (*h* ^2^: 0.12–0.15, Gauzere et al. 2016; *h* ^2^: 0.18–0.37, Westergren et al. 2023; *h* ^2^: 0.56–0.58, Kramer et al. 2008; *h* ^2^: 0.72; Frank et al. 2017; *h* ^2^: 0.84–0.92, Bontemps et al. 2016). As heritability is population‐ and environment‐specific, it is possible to have a wide variation in estimates. Notably, the first year of our study (2021) was marked by a delayed but then sudden start of spring. After a prolonged cold period in April, a strong increase in temperature led to an uncommonly synchronized leaf out pattern, resulting in different *h* ^2^‐values for the 2 years of the study. Additionally, differences in experimental conditions (e.g., field studies vs. common garden experiments) can lead to different heritability estimates, as field experiments often have a higher microenvironmental variation (Westergren et al. 2023). Nevertheless, spring phenology is a trait that generally exhibits a high heritability, underlining its evolutionary potential.

In contrast, autumn phenology in European beech consistently shows a lower heritability (*h* ^2^: 0.35) than spring phenology, as also shown in literature (*h* ^2^: 0.09; Westergren et al. 2023; *h* ^2^: 0.20; Gauzere et al. 2016; *h* ^2^: 0.35; Frank et al. 2017). While often overlooked, the relevance of autumn should not be neglected (Gallinat et al. 2015). Under recent climatic changes, the growing season of trees has been observed to expand towards autumn; however, the consequences for annual tree growth and other physiological or phenological traits are still not fully understood. Recent studies also suggest complex interactions between spring and autumn phenology, which can advance or delay each other's timing (Beil et al. 2021; Garrigues et al. 2023). Especially in cases of late spring frost, an increased productivity in autumn can partially compensate for the negative effects of frost damages (Zohner et al. 2019). Furthermore, the expansion of the growing season can have contrasting effects in the growth of roots or shoots, depending on whether the expansion takes place in spring or autumn, which further complicates the already complex trait interactions and their ecological consequences (Zohner et al. 2021).

The considerable heritability in spring and autumn phenology suggests that European beech has the potential to evolve its timing with changing environmental conditions, provided that the selective force is directed and effective. However, selection on phenology is unlikely to be persistently unidirectional and effective. Generally, it is assumed that spring phenology underlies a stabilizing selection, because a badly timed leaf out (too early or too late) would lead to potential frost damage or a shortened growing season (Gömöry and Paule 2011). However, observations from natural European beech populations indicate substantial variation even within single natural tree stands of European beech. For the source stand of this experiment, Malyshev et al. (2022) reported a median deviation from 3 to 10 days in leaf out date from the median tree over 12 years. This might hint towards fluctuating selection pressures, since different spring phenology strategies (early or late leaf out) could be advantageous, depending on the appearance of late frosts (Gömöry and Paule 2011). Despite climate change, late spring frost damages are expected to increase in future (Zohner et al. 2020). Especially in the case of expanding growing seasons and advancing spring phenology, European beech is therefore facing an increased risk of frost damage (Sangüesa‐Barreda et al. 2021). Thus, a well‐adjusted timing of leaf out date is critical for European beech to escape frost damage and maintain its fitness.

While the timing of spring and autumn phenology may evolve, the adaptive potential of frost tolerance is more questionable. Our results indicate that selective events would only have a small directing effect on the collective gene pool of the population because the observed frost tolerance is only weakly explained by underlying additive genetic variance. We found only a low heritability in frost tolerance of buds (*h* ^2^: 0.12) and unfolded leaves (*h* ^2^: 0.13). To our knowledge, no other studies have yet reported heritability estimates of frost tolerance in buds or leaves in European beech. Still, it has been shown that spring phenology and frost tolerance of leaves correlate well across woody species (Vitasse et al. 2014). For European beech, there are also studies that show that the species has experienced genetic differentiation in frost tolerance, implying that adaptive processes might indeed be possible in this trait (Gömöry et al. 2015; Kreyling et al. 2014). However, for an effective adaptation to occur, selective events need to lie within a “selective window”, the span in which some individuals face a fitness (dis‐)advantage while others do not. In our study, the span in the frost tolerance in freshly unfolded leaves of the most and the least tolerant individuals was small (average: −7.76°C ± 0.72 SD). Generally, frost tolerance of unfolding leaves of European beech is also known to be very limited (Menzel et al. 2015; Bianchi et al. 2019). Conclusively, a strong or critically timed late frost event might affect most individuals evenly. Taking the low heritability and inconsistent selection into account, we conclude that frost tolerance of leaves in spring might only adapt slowly. Nevertheless, our result does not contradict the possibility of adaptive processes; it only raises the question of how fast they could take place.

The importance of frost tolerance towards the end of the growing season in autumn has been studied even less than spring frost tolerance. The actual frequency and magnitude of early autumn frosts in the distribution range of European beech is still questionable. The few existing studies assume that an early frost would harm the nutrient retraction from leaves and would therefore affect the nutrient storage for the next year (Norby et al. 2003; Fracheboud et al. 2009; Hänninen et al. 1990; Estiarte and Peñuelas 2015). In contrast, buds are unlikely to be affected by early autumn frost, since they commonly exhibit a sufficiently deep frost tolerance (Kreyling et al. 2015).

Taken together, our results suggest that European beech has the genetic capacity to evolve its spring and autumn phenology and thereby adapt to changing environmental conditions. Potentially, the limited frost tolerance and the low capacity for adaptation in this trait could affect the extent to which spring and autumn phenology can advance, as the avoidance of frost damage remains crucial for a tree's fitness. In this context it is important to consider that the pace of adaptive processes of traits also depends on the strength of selection, which is not equal across traits (Kingsolver et al. 2001). Especially for long‐lived organisms like trees, selective events in the year of germination, such as drought, frost or pathogens, can be strong and lead to an increased mortality (Muffler et al. 2021). Once the tree is established, however, stressors merely affect the tree's reproductive success by reducing its investment of resources or by directly affecting the reproduction, for example, by frost damaging the flowers. In conclusion, it can be cumbersome to estimate and compare the adaptive pace of different traits. Assuming that the effect of selective events is strongest during the youth of a tree and becomes weaker once the tree reaches maturity and starts to reproduce, the pace of adaptation to a changing climate is generally slow for such long living species.

In general, frost avoidance by an adjusted and variable phenology within forest stands appears to be an important strategy of European beech to mitigate the risk of frost damage (Menzel et al. 2015; Lenz et al. 2016). Possibly, the evolution of frost tolerance and avoidance is closely connected to the ecological niche of the species. Baumgarten et al. (2023) also suggest that the recovery potential of a species might be a relevant factor for the adjustment of a species' spring phenology to frost risks. European beech is a dominant, shade‐tolerant tree that has a flexible and expansive biomass allocation pattern and an opportunistic strategy in utilizing its nonstructural carbon reserves (NSC) (Barbaroux et al. 2003; Petriţan et al. 2009; D'Andrea et al. 2021). It has a comparable frost tolerance to competing tree species; however, spring frost events can result in lagged recovery rates (Baumgarten et al. 2023). Late spring frost damages might have a high impact on European beech, as the formation of new leaves is very costly and depletes reserves of NSC more severely than those of competitors such as oak (Barbaroux et al. 2003). However, studies suggest that European beech directly recovers its growth rate in the year after the frost damage (Príncipe et al. 2017; Vander Mijnsbrugge et al. 2021). Nevertheless, the safest strategy is to avoid frost by a well‐timed phenology.

From an ecological perspective, a slow response of European beech to changing environmental conditions would not necessarily mean that European beech would face a critical environmental mismatch. However, if the speed of response would be too slow, the species or at least some of its populations might decline in their competitive advantage over other species that have a higher phenological plasticity and are more likely to benefit from an early onset of the growing season. Especially in the context of climate change leading to more frequent and severe summer droughts, it can be crucial to capitalize on the warmer conditions in early spring. In some climatic regions, various oak species, which are known to be more plastic in their timing of spring phenology, might gain ground in the direct competition with European beech (Vitasse et al. 2011; Penuelas and Boada 2003). Yet, caution is needed when extrapolating the results, as heritability is specific for a population and for an environment (Visscher et al. 2008). Furthermore, the interaction of phenology and frost tolerance is not thoroughly studied yet, and also other traits and mechanisms might be relevant, for example, the potential to cope with frost damages (resilience) (Príncipe et al. 2017; Zohner et al. 2019; Baumgarten et al. 2023). Nevertheless, this study sheds light on the potential interaction and adaptive capacity of the selected traits, implying that European beech has the phenotypic variation and the heritability to adapt the timing of its growing season, but that adaptation by evolution of frost tolerance is limited. Frost avoidance appears to be its main strategy to deal with late spring frost risk, since frost tolerance is either hardly evolving or physiologically restricted.

## Author Contributions

**Jonas Schmeddes:** conceptualization (equal), data curation (lead), formal analysis (equal), investigation (lead), methodology (equal), visualization (equal), writing – original draft (lead), writing – review and editing (equal). **Niels Preuk:** conceptualization (supporting), data curation (supporting), formal analysis (equal), investigation (supporting), methodology (equal), supervision (supporting), writing – original draft (supporting), writing – review and editing (supporting). **Ilka Beil:** data curation (supporting), investigation (supporting), methodology (supporting), supervision (supporting), writing – review and editing (supporting). **Eike Marie Dittmar:** data curation (supporting), investigation (supporting), writing – review and editing (supporting). **Andrey Malyshev:** funding acquisition (supporting), methodology (supporting), writing – review and editing (supporting). **Annè Lemke:** data curation (supporting), investigation (supporting), writing – review and editing (supporting). **Charlotte Loft:** data curation (supporting), investigation (supporting), writing – review and editing (supporting). **Dennis Quadt:** data curation (supporting), investigation (supporting), writing – review and editing (supporting). **Maximilian Richardt:** data curation (supporting), investigation (supporting), writing – review and editing (supporting). **Tom Woweries:** data curation (supporting), investigation (supporting), writing – review and editing (supporting). **Juergen Kreyling:** conceptualization (lead), funding acquisition (lead), investigation (supporting), methodology (supporting), project administration (lead), resources (lead), supervision (lead), validation (equal), visualization (supporting), writing – original draft (supporting), writing – review and editing (equal).

## Funding

The study was funded by the DFG (Deutsche Forschungsgemeinschaft) Research Training Group RESPONSE (DFG RTG 2010).

## Conflicts of Interest

The authors declare no conflicts of interest.

## Data Availability

The underlying data and R‐scripts are publicly and permanently available at https://ckan.fdm.uni‐greifswald.de/dataset/data‐to‐schmeddes‐et‐al‐fagus‐heritability.
